# Immunovirological and environmental screening reveals actionable risk factors for fatal COVID-19 during post-vaccination nursing home outbreaks

**DOI:** 10.1038/s43587-023-00421-1

**Published:** 2023-05-22

**Authors:** Lize Cuypers, Els Keyaerts, Samuel Leandro Hong, Sarah Gorissen, Soraya Maria Menezes, Marick Starick, Jan Van Elslande, Matthias Weemaes, Tony Wawina-Bokalanga, Joan Marti-Carreras, Bert Vanmechelen, Bram Van Holm, Mandy Bloemen, Jean-Michel Dogne, François Dufrasne, Keith Durkin, Jean Ruelle, Ricardo De Mendonca, Elke Wollants, Pieter Vermeersch, COVID-19 Genomics Belgium Consortium, Ruddy Wattiez, Michael Peeters, Kate Bakelants, Sarah Denayer, François E. Dufrasne, Cécile Meex, Laurent Gillet, Maria Artesi, Marie-Pierre Hayette, Sébastien Bontems, Vincent Bours, Claire Gourzonès, Olivier Ek, Fabrice Bureau, Benoit Kabamba, Jean-Luc Gala, Bertrand Bearzatto, Jérôme Ambroise, Arnaud Marchant, Coralie Henin, Benoit Haerlingen, Ricardo de Mendonca, Marie-Luce Delforge, Carl Vael, Lynsey Berckmans, Philippe Selhorst, Kevin K. Ariën, Sonia Van Dooren, Bruno Hinckel, Hideo Imamura, Toon Janssen, Ben Caljon, Oriane Soetens, Denis Piérard, Thomas Demuyser, Charlotte Michel, Olivier Vandenberg, Sigi van den Wijngaert, Giulia Zorzi, Philippe Van Lint, Walter Verstrepen, Reinout Naesens, Sarah Van Lent, Pascale Hilbert, Sylvain Brohée, Pierre-Emmanuel Léonard, Deniz Karadurmus, Jeremie Gras, Damien Féret, Barbara Lambert, Anne Vankeerberghen, Astrid Holderbeke, Hans De Beenhouwer, Lien Cattoir, Christine Lammens, Basil Britto Xavier, Marie Le Mercier, Jasmine Coppens, Veerle Matheeussen, Herman Goossens, Geert A. Martens, Koen Swaerts, Frederik Van Hoecke, Dieter Desmet, Patrick Descheemaeker, Pierre Bogaerts, Jonathan Degosserie, Olivier Denis, Te-Din Huang, Dagmar Obbels, Hanne Valgaeren, Johan Frans, Annick Smismans, Paul-Emile Claus, Denise Veltman, Truus Goegebuer, Ann Lemmens, Bea Van den Poel, Sonja De Bock, Wim Laffut, Ellen Van Even, Jos Van Acker, Charlotte Verfaillie, Elke Vanlaere, Klara De Rauw, Luc Waumans, Britt Van Meensel, Reinoud Cartuyvels, Marijke Raymaekers, Bruno Verhasselt, Jorn Hellemans, Merijn Vanhee, Marijke Reynders, Caroline Boulouffe, Achille Djiena, Caroline Broucke, Boudewijn Catry, Katrien Lagrou, Marc Van Ranst, Johan Neyts, Guy Baele, Piet Maes, Emmanuel André, Simon Dellicour, Johan Van Weyenbergh

**Affiliations:** 1 Department of Laboratory Medicine, National Reference Centre for Respiratory Pathogens, University Hospitals Leuven, Leuven, Belgium; 2 Department of Microbiology, Immunology and Transplantation, Laboratory of Clinical Microbiology, KU Leuven, Leuven, Belgium; 3 Department of Microbiology, Immunology and Transplantation, Laboratory of Clinical and Epidemiological Virology, Rega Institute, KU Leuven, Leuven, Belgium; 4 Department of Pharmacy, Namur Research Institute for Life Sciences, University of Namur, Namur, Belgium; 5 Laboratory of Proteomics and Microbiology, University of Mons, Mons, Belgium; 6 Department of Infectious Diseases, Laboratory of Viral Diseases, National Institute for Public Health (Sciensano), Brussels, Belgium; 7 Laboratory of Human Genetics, GIGA Research Institute, Liège, Belgium; 8 Medical Microbiology Unit (MBLG), Institute of Experimental and Clinical Research (IREC), Université Catholique de Louvain, Brussels, Belgium; 9 Université Libre de Bruxelles (ULB), Brussels, Belgium; 10 Infectious Disease Surveillance Unit, Agence pour une vie de qualité (AVIQ), Wallonia, Belgium; 11 Outbreak Investigation Team, Agentschap zorg en gezondheid, Flanders, Belgium; 12 Unit Healthcare-Associated Infections and Antimicrobial Resistance, Sciensano, Brussels, Belgium; 13 Department of Microbiology, Immunology and Transplantation, Laboratory Virology and Chemotherapy, Rega Institute, KU Leuven, Leuven, Belgium; 14 Spatial Epidemiology Lab (SpELL), Université Libre de Bruxelles, Bruxelles, Belgium; 15 Department of Microbioloy, KLINA Hospital, Brasschaat, Belgium; 16 Virology Unit, Department of Biomedical Sciences, Institute of Tropical Medicine Antwerp & Department of Biomedical Sciences, University of Antwerp, Antwerp, Belgium; 17 Department of Microbiology, UZ Brussel, Vrije Universiteit Brussel, Brussels, Belgium; 18 Department of Microbiology, Laboratoire Hospitalier Universitaire de Bruxelles – Universitair Laboratorium Brussel (LHUB-ULB), Brussels, Belgium; 19 Clinical Laboratory, Department of Molecular Diagnostics, GZA Hospitals, Wilrijk, Belgium; 20 Department of Medical Microbiology, ZiekenhuisNetwerk Antwerpen (ZNA), Antwerp, Belgium; 21 Department of Molecular Biology, Institute of Pathology and Genetics (IPG) ASBL, Gosselies, Belgium; 22 Clinical Laboratory of Microbiology, OLVZ Aalst, Aalst, Belgium; 23 Department of Microbiology, University Hospital Antwerp, Edegem & Laboratory of Medical Microbiology, Vaccine & Infectious Disease Institute (VAXINFECTIO), University of Antwerp, Wilrijk, Belgium; 24 Department of Laboratory Medicine, AZ Delta General Hospital, Roeselare, Belgium; 25 Department of Laboratory Medicine, CHU UCL Namur, Yvoir, Belgium; 26 Briant Network, Department of Medical Microbiology, Imelda Hospital, Bonheiden, Belgium; 27 Department of Clinical Microbiology, AZ St Maarten, Mechelen, Belgium; 28 Department of Microbiology, AZ Jan Portaels, Vilvoorde, Belgium; 29 Department of Microbiology, Heilig Hart hospital Lier, Lier, Belgium; 30 General Hospital AZ St Lucas, Ghent, Belgium; 31 Department of Clinical Biology, Jessa Hospital, Hasselt, Belgium; 32 Department of Diagnostic Sciences, Ghent University Hospital, Ghent University, Ghent, Belgium; 33 Department of Laboratory Medicine, Medical Microbiology, AZ St-Jan Brugge-Oostende AV, Brugge, Belgium

**Keywords:** Predictive markers, Infectious diseases, Ageing, Gene expression analysis, SARS-CoV-2

## Abstract

Coronavirus Disease 2019 (COVID-19) vaccination has resulted in excellent protection against fatal disease, including in older adults. However, risk factors for post-vaccination fatal COVID-19 are largely unknown. We comprehensively studied three large nursing home outbreaks (20–35% fatal cases among residents) by combining severe acute respiratory syndrome coronavirus 2 (SARS-CoV-2) aerosol monitoring, whole-genome phylogenetic analysis and immunovirological profiling of nasal mucosa by digital nCounter transcriptomics. Phylogenetic investigations indicated that each outbreak stemmed from a single introduction event, although with different variants (Delta, Gamma and Mu). SARS-CoV-2 was detected in aerosol samples up to 52 d after the initial infection. Combining demographic, immune and viral parameters, the best predictive models for mortality comprised *IFNB1* or age, viral *ORF7a* and *ACE2* receptor transcripts. Comparison with published pre-vaccine fatal COVID-19 transcriptomic and genomic signatures uncovered a unique *IRF3* low/*IRF7* high immune signature in post-vaccine fatal COVID-19 outbreaks. A multi-layered strategy, including environmental sampling, immunomonitoring and early antiviral therapy, should be considered to prevent post-vaccination COVID-19 mortality in nursing homes.

## Abstract (web summary)

Although vaccination drops COVID-19 mortality in older adults, post-vaccine fatal COVID-19 in nursing home outbreaks was linked to Delta, Gamma and Mu variants, persistently detected in aerosols. Mortality was predicted by IFNB1 or age, ORF7a and ACE2 mRNAs.

## Main

Severe acute respiratory syndrome coronavirus 2 (SARS-CoV-2) outbreaks affecting nursing homes have been a major public health concern since the start of the Coronavirus Disease 2019 (COVID-19) pandemic. During the first epidemic wave, it was estimated that COVID-19 mortality in Belgium was up to 130 times higher inside than outside nursing homes, due to the combined effects of age, sex, frailty and infection risks among residents^1^. Spatial analyses also indicated an association between the hospitalization incidence and the local density of nursing home residents, thus confirming the important impact of COVID-19 outbreaks in those facilities^2^. With one of the highest documented COVID-19 mortality rates in the world^2^, more than half of all COVID-19-related deaths in 2020 in Belgium were linked to nursing homes^3^. A meta-analysis of the first COVID-19 wave in Spain found that mortality at the facility level was significantly associated with a higher percentage of patients with complex diseases, lower scores on pandemic preparedness measures and higher population incidence of COVID-19 in the surrounding population^4^.

Nursing home residents are usually characterized by advanced age, a wide arsenal of comorbidities and associated polypharmacy and a decreased function of the immune system, potentially resulting in a higher risk of infections^4–8^. To protect this highly vulnerable population, the rollout of the vaccination campaign was initially targeted toward older adults and healthcare workers. Vaccination in Belgian nursing homes began in the second half of December 2020, employing mainly the mRNA vaccine BNT162b2. The BNT162b2 vaccine is highly effective at protecting against COVID-19 hospitalization and death, with efficacies of 90–95% reported in phase 3 clinical trials^9^ and confirmed in large-scale real-life studies^10^. By March 2021, vaccination coverage (two-dose scheme) among residents of nursing homes had reached 89.4% on a national scale. Starting from September 2021 on, a third or booster dose was administered in nursing homes. Reduction in hospital admissions and mortality among residents of nursing homes on account of vaccination has been reported throughout Europe, such as for a Spanish study that included over 25,000 residents and reported a fatality rate of only 1.6% in the post-vaccination era^11^. A recent study of 10 European countries, analyzing 240 COVID-19 outbreaks in the post-vaccination era (July–October 2021), identified an average case fatality rate of 5.5% for Belgium, almost half of the European average of 10.2%^12^. Although the same study identified vaccination status as significantly associated with COVID-19 hospitalization, no association was found with COVID-19 mortality. Strong variability in case fatality ratios has been observed^13–15^, with no major risk factors of fatal post-vaccination COVD-19 identified so far, other than age and comorbidities, mostly due to the limited statistical power in small outbreaks.

Through our nationwide surveillance, we observed only three high fatality rate (>10%) post-vaccination outbreaks in Belgian nursing homes by the end of this study (October 2021). Here we describe a multidisciplinary investigation of these three post-vaccination outbreaks in a collaboration involving the nursing home staff, health inspectors of the respective regional agencies, the national institute for public health (Sciensano), political, academic and governmental stakeholders as well as the National Reference Center of Respiratory Pathogens at the University Hospital and University of Leuven. Thus, we were able to identify demographic and clinical risk factors as well as a unique prognostic gene signature for fatal COVID-19 in vaccinated nursing home residents, revealing actionable public health and precision medicine strategies to mitigate COVID-19 mortality among susceptible older adults in the post-vaccine era.

## Results

### Epidemiological profile of SARS-CoV-2 nursing home outbreaks

**Fig. 1:**
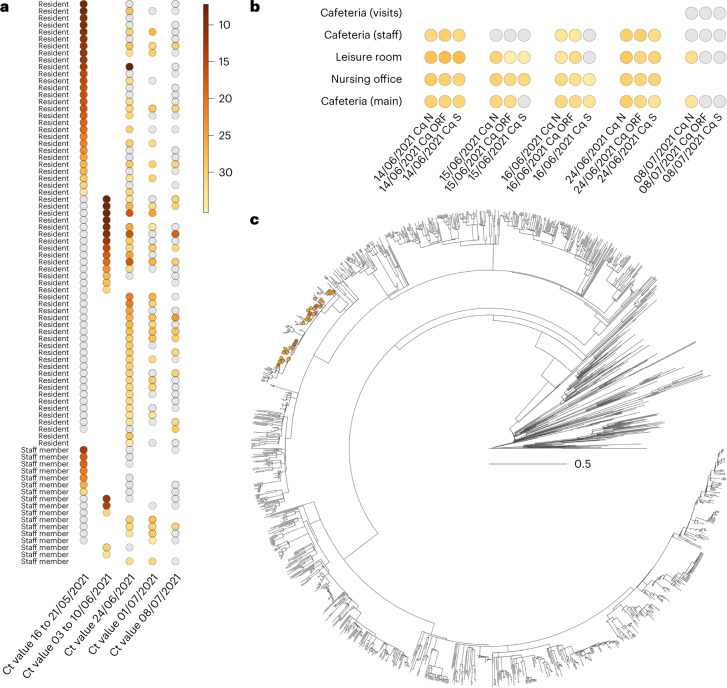
Overview of the outbreak in nursing home A (Delta/B.1.617.2). **a**,**b**, We report the evolution through time of Ct values measured in both infected residents and staff members (**a**) and aerosols analyzed in various sections within the nursing home (**b**). Gray dots refer to negative PCR results. **c**, Time-scaled phylogenetic analysis involving Delta (B.1.617.2) genomes sampled and sequenced from this outbreak reveals that all 58 full genomes originating from nursing home A are clearly clustered within the overall phylogenetic tree (orange dots), suggesting a single introduction event. The phylogenetic tree is time calibrated, meaning that branch lengths are in units of time (year).

For the largest of the three outbreaks (nursing home A), the first infection was documented in the dementia ward on 17 May 2021, for an 89-year-old woman who developed COVID-19-related symptoms, who was subsequently hospitalized and who died after 2 weeks of hospitalization. A total of 102 cases were documented related to this outbreak between 18 May and 24 June, of which 75 were residents, 25 were staff members and two were family members of staff. All departments of the nursing home were involved, and consecutive screening moments were scheduled. Among 120 residents, 75 were SARS-CoV-2 positive by polymerase chain reaction (PCR) (62.5%; Table 1), whereas only 25 of 146 (17.1%) staff members tested positive (Supplementary Table 1). Timing of diagnosis by a positive PCR result and longitudinal follow-up is illustrated in Fig. 1a, which clearly shows late-onset PCR positivity for a large subset of residents who tested PCR negative at the start of the outbreak. This ‘second wave’ of delayed infections was corroborated by the continuous detection of SARS-CoV-2 by quantitative PCR (qPCR) in aerosol samples taken from the common areas of both staff and residents (Fig. 1b). For 58 of 102 (56.9%) positive cases, whole-genome sequencing (WGS) information was available, identifying the Delta variant (Pangolin lineage B.1.617.2) for all of them. Phylogenetic analysis indicates that all samples from the nursing home cluster were within the same clade, hence suggesting a single introduction event (Fig. 1c). Among the 75 PCR-positive residents, 15 died (case fatality ratio of 20%). Considering all individuals for whom vaccination status was known (Table 1), 96% of residents, but only 66% of staff members, were fully vaccinated. One resident and five staff members were partially vaccinated at the time of the outbreak, whereas one resident and 28.7% of staff members were not vaccinated.

**Table 1:** Demographic and clinical characteristics of nursing home residents involved in the three post-vaccination outbreaks

Characteristics	Nursing home A Delta	Nursing home B Gamma	Nursing home C Mu^a^
Median age, years (range)	87 (63–102)	82 (59–98)	87 (64–103)
Sex, number (%)			
Male	71 (26.5%)	11 (23.9%)	77 (25.2%)
Female	197 (73.5%)	35 (76.1%)	229 (74.8%)
Start vaccinating residents	8 Jan 2021	12 Jan 2021	26 Jan 2021
Vaccination ratio (among PCR+)			
2 doses	96% (94.6%)	86.2% (89.5%)	98.0% (100%)
1 dose	1% (1.4%)	13.0% (10.5%)	0.4% (0%)
0 doses	3% (4.1%)	0%	1.3% (0%)
First documented case	17 May 2021	20 May 2021	20 July 2021
PCR positivity	62.5% (75/120)	65.5% (19/29)	12.0% (20/166) 69.0% (20/29)^d^
Case fatality ratio (only PCR+)	20.0% (15/75)^b^	31.6% (6/19)^c^	35.0% (7/20)^b^

^a^For nursing home C, three isolated Delta cases were observed in addition to the Mu outbreak. All residents received the Comirnaty (Pfizer) vaccine.

^b^An additional resident died, not SARS-CoV-2 PCR positive, with death considered not COVID-19 related.

^c^A total of seven fatal cases, of which one was not SARS-CoV-2 PCR positive; this death was classified as COVID-19 related due to severe respiratory symptoms and recent close contact with positive residents, according to WHO criteria^16^.

^d^Considering only the 29 residents of the two affected wards, positivity rates increase up to 69.0%.

**Extended Data Fig. 1:**
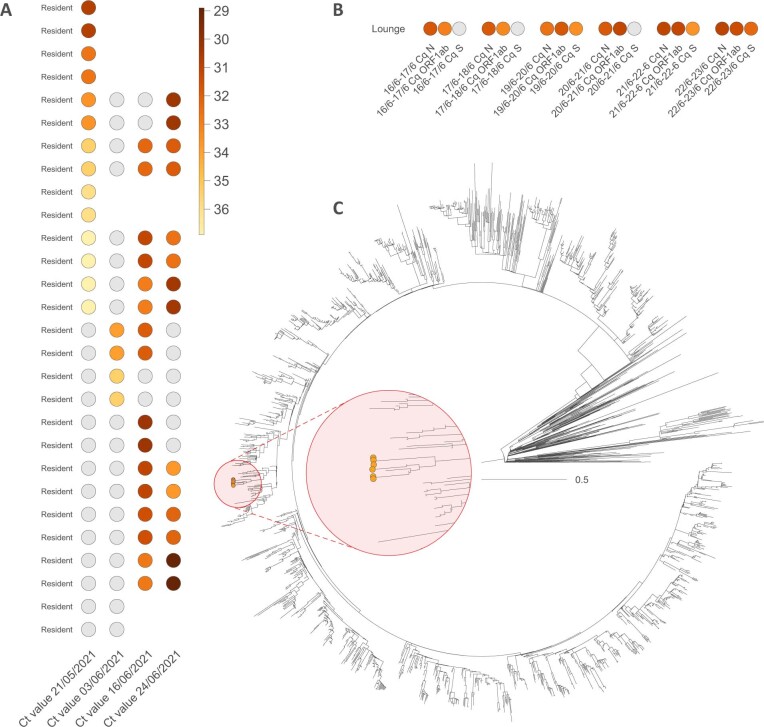
Overview of the outbreak in nursing home B (Gamma/P.1). We report the evolution through time of Ct values measured in both infected residents (**a**) and aerosols analyzed in the lounge of the nursing home (**b**). Grey dots refer to negative PCR results. In addition, we also report the time-scaled phylogenetic analysis involving Gamma (P.1) genomes sampled and sequenced from this outbreak (**c**), showing one phylogenetic cluster (zoomed in the red circle, scale bar corresponds to the full tree) among 6 full genomes (orange dots), likely corresponding to a single introduction event into nursing home B. The phylogenetic tree is time-calibrated, meaning that branch lengths are in units of time (year).

The first documented PCR-positive case for nursing home B dates from 20 May 2021, and the presumed index case developed COVID-19 symptoms the day before. Overall, 19 of 29 residents (65.5%) tested positive for SARS-CoV-2, but none of the 17 staff members tested positive on the repetitive screening moments organized between 20 May and 24 June. Despite high cycle threshold (Ct) values for this outbreak (Extended Data Fig. 1), WGS was successful for 19 of 19 (100%) PCR-positive cases, all classified as Gamma variant (Pangolin lineage P.1). Our phylogenetic analysis highlights that all samples clustered together within the more global Gamma phylogeny inferred in our study, again pointing toward the hypothesis of a single introduction event (Extended Data Fig. 1). Overall, seven fatal cases were reported in this outbreak, of which one resident tested negative by PCR. Although this death was classified as COVID-19 related according to World Health Organization (WHO) criteria^16^, due to severe respiratory symptoms and recent close contact with positive residents, we conservatively used only PCR-positive residents to calculate the case fatality ratio (6/19, 32%). For this nursing home, the vaccination rate was high among residents (86.2%), whereas only 52.9% of the staff members were fully vaccinated at the time of the outbreak. Nevertheless, none of the latter tested positive for SARS-CoV-2.

**Extended Data Fig. 2:**
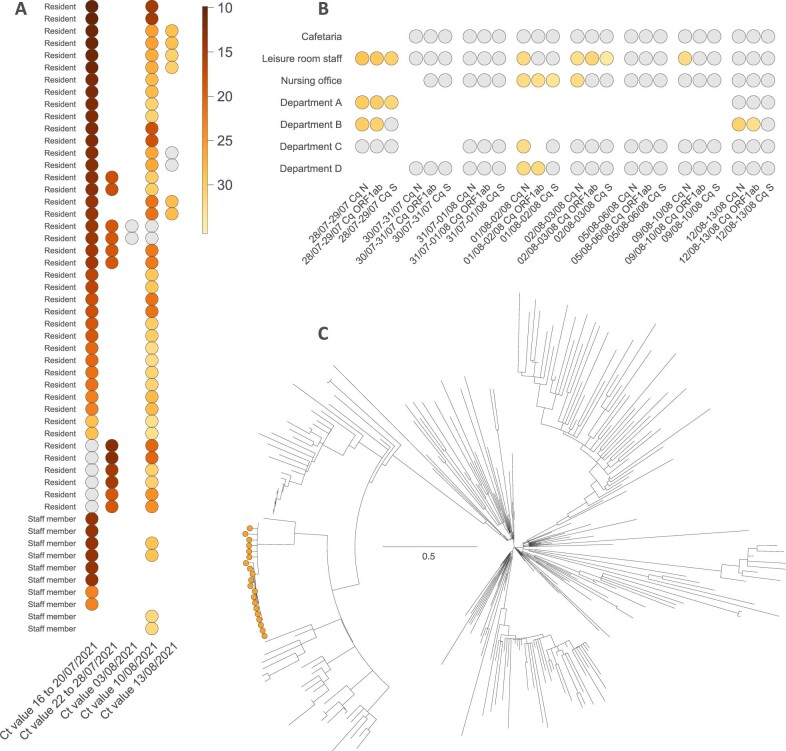
Overview of the outbreak in nursing home C (Mu/B.1.621). We report the evolution through time of Ct values measured in both infected resident/staff members (**a**) and aerosols analyzed in various sections within the nursing home (**b**). Grey dots refer to negative PCR results. In addition, we also report the time-scaled phylogenetic analysis involving Mu (B.1.621) genomes sampled and sequenced from this outbreak (**c**), showing one phylogenetic cluster (with short branch lengths) among 24 full genomes (orange dots) likely corresponding to a single introduction event. The phylogenetic tree is time-calibrated, meaning that branch lengths are in units of time (year).

The post-vaccination outbreak in nursing home C was initially alerted by two cases (related resident and staff) infected with the Delta variant a few days before the large testing initiative for the other residents and staff members (20 July 2021). Twenty-five additional SARS-CoV-2-positive cases were identified during the outbreak. WGS determined the presence of the variant of interest, Mu (Pangolin lineage B.1.621), complemented with the mutation K417N in the spike protein, and, for one isolated staff member without resident contact, an additional Delta infection was identified. The single Delta-infected resident was, therefore, not included for further analysis of the outbreak (Table 1; 27/27 PCR-positive cases (100%) were confirmed by WGS: three Delta and 24 Mu). The Mu variant saw relatively limited circulation in Belgium, resulting in a restricted sampling of related genomic sequences in the local community. Our phylogenetic analysis, however, indicates that PCR-positive cases in this nursing home related to that variant clearly clustered within the overall phylogeny inferred for that variant (Extended Data Fig. 2), again advocating for a single introduction event. Moreover, all 24 PCR-positive cases infected with variant Mu (20 residents and four staff members) were linked to the dementia unit of the nursing home. Overall, seven infected residents died of COVID-19 (7/20, case fatality ratio 35%), and one additional resident died of a COVID-19-unrelated cause. Considering the 229 residents and staff members with known vaccination status, the overall vaccination rate was 98.3%. For the group of PCR-positive residents, 100% were fully vaccinated.

### Demographic and clinical profile of SARS-CoV-2 outbreaks

**Extended Data Fig. 3:**
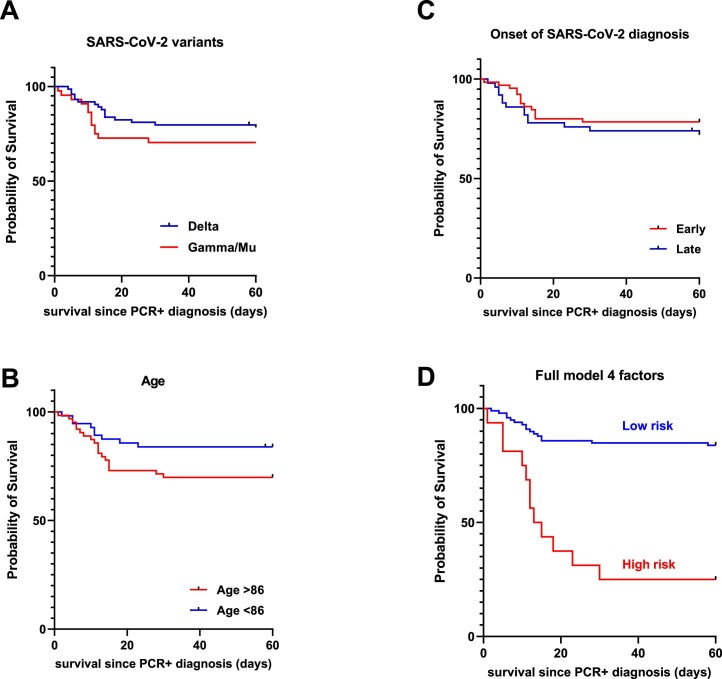
Univariate and multivariate Kaplan-Meier survival curves. Kaplan-Meier survival curves comparing (**a**) SARS-CoV-2 variants Delta vs. non-Delta (Gamma/Mu, Log-rank test, *p* = 0.28), (**b**) Age above or below the median (86 years, Log-rank test, *p* = 0.078); (**c**) Onset of SARS-CoV-2 diagnosis (PCR+): early (0–7 days) vs. late (>7 days), with regard to the start of the respective outbreaks, Log-rank test, *p* = 0.40; (**d**) Combined probability of age, sex, variant, and late onset of diagnosis (Log-rank test, p = 1.1 × 10^−7^). Source data

Demographic and clinical risk factors for fatal COVID-19 among residents were identified by multivariable logistic regression models (Table 2), with the best model including age, male sex, non-Delta SARS-CoV-2 variants (Gamma and Mu) and later onset of infection (PCR positivity >7 d after the start of the outbreak). In the sensitivity analysis, only fully vaccinated and PCR-positive residents (*n* = 107) were included. The results remained statistically significant, with a similar effect size (Supplementary Table 2). The importance of these four factors as predictors of mortality was confirmed by Kaplan–Meier survival estimates (Extended Data Fig. 3) and time-to-event analysis (Cox proportional hazard regression; Supplementary Table 3). Of interest, dementia or peak viral load (nadir cycle quantification (Cq) value) were not predictive of fatal cases in the joint analysis of the three outbreaks (Table 2) but were significant predictors in single nursing homes (Supplementary Table 3). Because nursing home size was found to be a major risk factor for COVID-19 mortality in several countries, including Belgium^17,18^, we included this as an additional parameter in both logistic and Cox regression models. As shown in Supplementary Table 6, nursing home size was not an independent predictor (in addition to age, sex and late PCR positive) of fatal COVID-19, whereas the preferred model (corrected Akaike information criterion (cAIC)) contained age, sex, late PCR positive and variants/outbreaks as independent predictors.

**Table 2:** Multivariate logistic regression of demographic and clinical characteristics of residents with COVID-19 (all PCR-positive residents, *n* = 114)

	Model 1		Model 2		Model 3^a^		Model 4	
Variable	Odds ratio	95% CI	Odds ratio	95% CI	Odds ratio	95% CI	Odds ratio	95% CI
**Sex (M)**	**3.38**	**1.24–9.47**	**3.55**	**1.29–10.1**	**6.03**	**1.91–21.21**	**5.68**	**1.72–20.94**
**Age**	**1.08**	**1.02–1.15**	**1.08**	**1.02–1.16**	**1.13**	**1.05–1.22**	**1.15**	**1.06–1.25**
**SARS-CoV-2 Gamma/Mu**	−	−	1.74	0.71–4.32	**3.97**	**1.26–13.98**	**3.73**	**1.14–13.62**
**Late PCR+**	−	−	−	−	**3.28**	**1.04−11.58**	2.96	0.92–10.72
Dementia	−	−	−	−	−	−	0.99	0.36–2.76
Diabetes	−	−	−	−	−	−	1.44	0.39–5.10
Nadir Cq value	−	−	−	−	−	−	1.01	0.95–1.06

^a^Model 3 was the best model, according to cAIC; significant variables are indicated in bold. Late PCR+, late onset of PCR positivity (≤7 d versus >7 d after first PCR-positive case in each nursing home).

### Digital transcriptomic analysis of SARS-CoV-2 outbreaks

In search of candidate biomarkers for post-vaccine fatal COVID-19, as well as possible therapeutic targets, we opted for nCounter digital transcriptomics for immunovirological profiling of the nasal mucosa, encouraged by previous results^19–21^. For 20 of 28 fatal cases, a sufficient volume of diagnostic nasopharyngeal swabs was available for nCounter analysis, to explore immunological (600 genes representative of the major immune cell types) and virological (SARS-CoV-2 transcripts and ACE2/TMPRSS2 receptors) parameters as possible risk factors for fatal post-vaccine COVID-19. Thus, we carefully matched (age, sex and outbreak) 20 fatal cases (all those with available nasopharyngeal swabs) with 30 PCR-positive non-fatal cases, with similar timing of infection, as well as 10 PCR-negative but SARS-CoV-2-exposed residents. Because these samples were obtained at SARS-CoV-2 diagnosis, before hospitalization or treatment (oxygen and/or dexamethasone), the transcriptomic immune signatures are not modified by immunomodulatory treatment and can be used to predict fatal outcome. In addition, only four of 118 PCR-positive residents had received corticosteroids before their SARS-CoV-2 diagnosis. None of them was a fatal case, and they were not included for nCounter analysis.

**Fig. 2:**
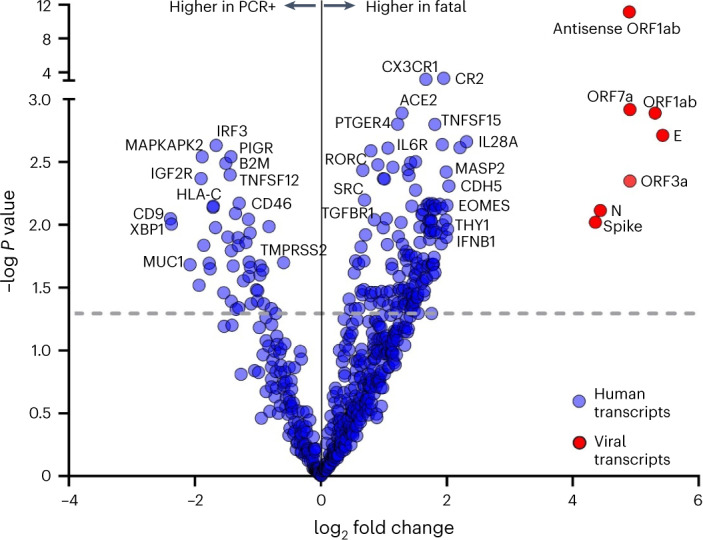
Differentially expressed genes in nasal mucosa of fatal COVID-19 outbreak cases as compared to matched PCR-positive residents from three nursing homes. Volcano plot of differentially expressed genes in nasal mucosa of fatal (*n* = 20) versus age-matched, sex-matched and outbreak-matched non-fatal PCR-positive cases (*n* = 30), quantified by nCounter digital transcriptomics (uncorrected *P* values from linear model, negative binomial distribution, dotted line showing *P* < 0.05, FDR *q* values provided in Source Data). Selected viral (red circles) and host immune transcripts (blue circles) significantly upregulated or downregulated in fatal versus non-fatal cases are highlighted with gene names. Details on immune genes are given in the Results section. PCR+, PCR positive. Source data

As shown in Fig. 2 (volcano plot), a total of 193 human and seven viral gene transcripts were significantly upregulated or downregulated (*P* < 0.05) when comparing fatal versus non-fatal cases. In addition to the antiviral cytokines *IL28A* (also known as *IFNL2* (interferon-λ2)) and *IFNB1* (the gene encoding interferon-beta (IFN-β)), the most upregulated genes were predominantly expressed by innate immune cells: monocytes/macrophages (*CX3CR1*, *TNFSF15*, *CLEC6A*, *ITLN1* and *LILRB5*), natural killer (NK) cells (*THY1*, *CDH5*, *KIR3DL3*, *CD160*, *B3GAT1*, *NCAM1* and *CCL3*) and conventional dendritic cells (*XCR1*). Thus, the predominant immunopathogenic signature of fatal COVID-19 in vaccinated residents represents exacerbated innate immune activation rather than a failed adaptive (B cell and T cell) vaccine response. Likewise, a large subset of B cell genes (*CD19*, *CR2*, *CD79A*, *CD79B*, *PAX5* and *CD70)*, regulatory T cell (Treg) genes (*FOXP3* and *PTGER4*) and cytotoxic CD8 T cell genes (*EOMES* and *PTGER4*) were also significantly upregulated in fatal cases, arguing against a curtailed B cell or T cell response or a failure of B cells or T cells to migrate to the nasal mucosa. On the other hand, a generalized downregulation of major histocompatibility complex (MHC) class I-mediated antigen presentation (*B2M* and *HLA-C*) was observed across all cell types, in agreement with previous reports demonstrating loss of MHC class I activity at the transcriptomic, epigenomic and functional level^22–27^.

**Fig. 3:**
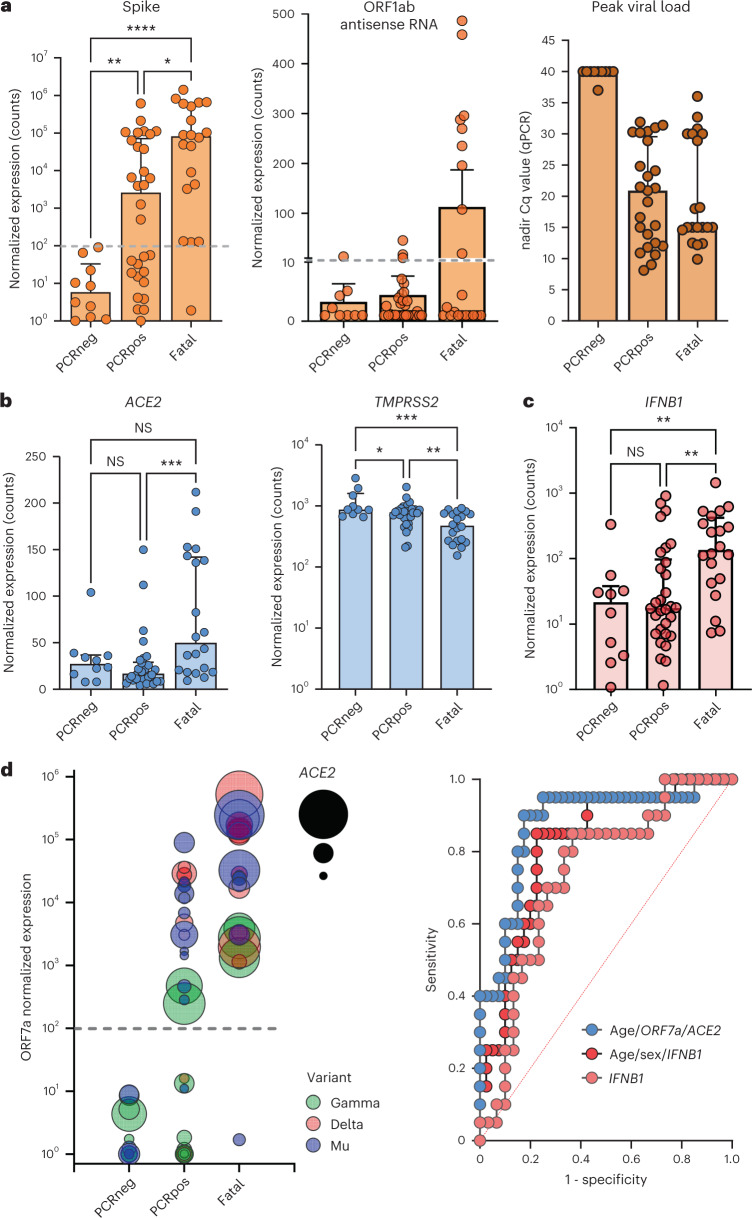
Immunological and virological risk factors identified in fatal COVID-19 outbreak cases among residents in three nursing homes. **a**–**c**, Viral transcript levels for spike protein (left: fatal versus PCRpos *P* = 0.012, fatal versus PCRneg *P* = 0.000022, PCRneg versus PCRpos *P* = 0.0089) and ORF1ab antisense RNA (middle), measured by nCounter digital transcriptomics. Right panel shows peak viral load (nadir Cq values) as quantified by qPCR. Viral receptors (*ACE2:* fatal versus PCRpos *P* = 0.0009*; TMPRSS2:* fatal versus PCRpos *P* = 0.0036, fatal versus PCRneg *P* = 0.0005, PCRneg versus PCRpos *P* = 0.0422) (**b**) and antiviral cytokine *IFNB1* (fatal versus PCRpos *P* = 0.0022, fatal versus PCRneg *P* = 0.0022) (**c**) were quantified by nCounter digital transcriptomics. Data are presented as median values ± s.d. **d**, Left: visualization of best predictive model (multivariate logistic regression, selected by cAIC), including age (not depicted) and *ORF7a* and *ACE2* transcripts. Dashed gray lines indicate the detection limit of SARS-CoV-2 transcripts. Each circle represents a resident, and the size of the circle is proportional to *ACE2* normalized expression. Right: comparison of ROC curves of predictive models by univariate (*IFNB1*) or multivariate (*IFNB1*/age/sex and age/*ORF7a/ACE2*) logistic regression. ROC curves showing significant prediction of fatal versus non-fatal COVID-19 according to *IFNB1* transcript levels (right), with and without age and sex as additional factors (detailed in the Results section). For **a**–**c**, statistical results are from Kruskal–Wallis test with FDR correction for multiple testing (PCRneg *n* = 10, PCRpos *n* = 30, fatal *n* = 20), ****P < 0.0001, ***P < 0.001, **P < 0.01, *P < 0.05, NS, not significant. PCRpos, PCR positive; PCRneg, PCR negative. Source data

Because the top downregulated genes were most representative of mucosal epithelial cells (*PIGR*, *CD9* and *MUC1*), the observed exacerbated innate response might represent enhanced migration of innate immune cells but also virus-mediated destruction of the mucosal epithelial cells. In favor of the latter hypothesis, fatal cases were characterized by significantly higher viral transcript levels when measured by nCounter. Transcript levels for spike, envelope, nucleoprotein, ORF1ab, ORF3a and ORF7a genes (Fig. 3a and data not shown, all *P* < 0.05 with false discovery rate (FDR) correction) were higher in fatal cases compared to non-fatal PCR-positive residents. In addition, antisense SARS-CoV-2 was selectively increased in eight of 20 fatal cases (Fig. 3a) versus PCR-positive cases, indicating heightened intracellular viral replication. Of note, peak viral load (nadir Cq values) or viral load of the first PCR-positive sample, measured by qPCR, was not significantly different between fatal cases and PCR-positive controls (Fig. 3a), underscoring the sensitivity of nCounter digital transcriptomics. Exacerbated viral replication in fatal cases was paralleled by a marked eight-fold increase in viral receptor *ACE2* transcript levels (*P* < 0.001) as well as an unexpected two-fold decrease (*P* < 0.01) in viral co-receptor *TMPRSS2* expression (Fig. 3b).

Among all immune genes, *IFNB1* transcripts displayed the strongest negative correlation to survival time (starting from the date of PCR-positive diagnosis, Spearman’s ρ = −0.24, *P* = 0.0024). Corroborating our previous findings in a Belgian cohort of intensive care unit (ICU) patients^19^, we found that increased *IFNB1* transcript levels significantly predicted a fatal outcome (Fig. 3c,d; area under the receiver operating characteristic (AUROC) curve 0.76 (95% confidence interval (CI) 0.63–0.89), *P* = 0.0013), which was slightly increased by adding age and sex to the model (Fig. 3d; AUROC 0.82 (95% CI 0.71–0.93), *P* = 0.000064). *IFNB1* remained a significant predictor in multivariable logistic regression, independent of age, sex and peak viral load (nadir Cq value), which was also confirmed by time-to-event analysis (Cox proportional hazard models; Table 3).

**Table 3:** Multivariate Cox proportional hazard regression of immunological and virological parameters in fatal versus non-fatal post-vaccination COVID-19 in nursing home residents

	Model 1^a^ (50 residents)		Model 2 (50 residents)	
Variable	Hazard ratio	95% CI	Hazard ratio	95% CI
Sex (M)	1.97	0.64–5.45	2.02	0.65–5.83
**Age**	**1.08**	**1.02–1.15**	**1.08**	**1.02–1.16**
***IFNB1*** **transcript** **levels** **(log)**	**2.32**	**1.26–4.48**	**2.36**	**1.26–4.63**
Nadir Cq value	−	−	1.01	0.95–1.06

^a^Model 1 was the best model according to cAIC; significant variables are indicated in bold.

Lastly, when combining all available demographic, immune and viral parameters, the best predictive model for mortality, according to the cAIC, included age (odds ratio (OR) 1.07, 95% CI 0.98–1.19), increased viral *ORF7a* (OR 1.67, 95% CI 0.98–3.46) and viral receptor *ACE2* (15.43, 95% CI 2.54–165.9) transcript levels, resulting in correct classification of 18 of 20 (90%) fatal cases (AUROC 0.88, 95% CI 078–0.98, *P* = 0.000002), as visualized in Fig. 3d.

### A unique immune signature in post-vaccination fatal COVID-19

To our knowledge, no well-powered study of immune signatures in post-vaccination fatal COVID-19 in the older adult population have been published at present. Thus, no public datasets are currently available for independent validation of our ‘post-vaccine fatal COVID-19’ immune signature in a comparable epidemiological setting. Therefore, we compared published transcriptomic and genomic signatures of pre-vaccination fatal and/or life-threatening COVID-19.

**Fig. 4:**
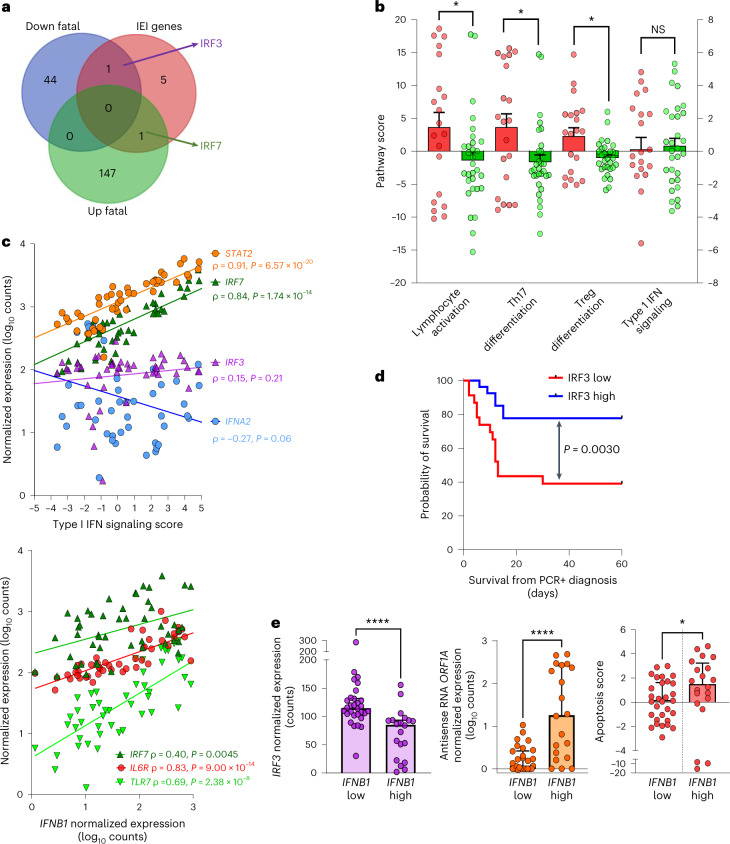
IRF3/IRF7 dichotomy in type I IFN signaling underlies IFN-β link to inflammation, apoptosis and mortality in nursing home residents during post-vaccine COVID-19 outbreaks. **a**, Venn diagram shows overlap between gene transcripts upregulated (‘up Fatal’) or downregulated (‘down Fatal) in fatal cases versus PCR-positive controls (quantified by nCounter digital transcriptomics) and the gene mutations (IEI) identified in life-threatening COVID-19 (ref. ^28^) (pre-vaccine era). The five IEI genes not differentially expressed between cases and controls are *TICAM1*, *TBK1*, *UNC93B1*, *IFNAR1* and *TLR3*. **b**, Pathway scores (calculated by nSolver from gene expression profiling by nCounter) for lymphocyte activation (*P* = 0.043), Th17 (*P* = 0.028) and Treg differentiation (*P* = 0.022) were increased in fatal cases versus PCR-positive controls, whereas type I IFN signaling was not (*t*-test with Welch’s correction). No pathways were significant after stringent Bonferroni correction for multiple testing. Data are presented as median values ± s.d. Red circles: fatal cases; green circles: PCR-positive controls. **c**, Spearmanʼs correlation of type I signaling score (upper panel) and *IFNB1* expression (lower panel) with drivers of IFN signaling (*STAT2*, *IRF7*, *IRF3, IFNA2* and *TLR7*) and inflammation (*IL6R*), across all 50 residents (20 fatal cases and 30 PCR-positive controls). **d**, Kaplan–Meier curve demonstrating significantly lower (log-rank test) survival in nursing home residents with ‘*IRF3* low’ status (nCounter normalized expression below the median). **e**, Classification of nursing home residents into ‘*IFNB1* high’ versus ‘*IFNB1* low’ (below or above 100 normalized counts) reveals a significant link with *IRF3* expression (Mann–Whitney test *P* = 0.000011), intracellular viral replication (measured as SARS-CoV-2 antisense RNA, Mann–Whitney test *P* = 0.000072) and apoptosis score (calculated by nSolver, Mann–Whitney test *P* = 0.044) in upper airway mucosa. Data are presented as median values ± s.d. ****P < 0.0001, **P < 0.01, *P < 0.05, NS, not significant. PCR+, PCR positive. Source data

Upon cross-comparison of our ‘post-vaccine fatal COVID-19’ transcriptomic signature with previously described IEI (inborn errors in type I IFN immunity) genes linked to life-threatening COVID-19 (ref. ^28^), we identified a clear dichotomy between upregulated (*IRF7*) versus downregulated (*IRF3*) IEI genes in fatal cases (Fig. 4a). Therefore, we quantified type I IFN signaling score based on nCounter gene expression data (Supplementary Table 5). Of note, type I IFN signaling score was not significantly different between fatal cases and matched controls (Fig. 4b). However, lymphocyte activation, Th17 and Treg differentiation pathways were significantly increased in fatal cases (Fig. 4b), in agreement with our finding of upregulated *EOMES*, *SRC*, *THY1*, *RORC*, *IL6R*, *FOXP3* and *PTGER4* genes (Fig. 2). As shown in Fig. 4c, type I IFN signaling score was highly correlated to *IRF7* (ρ = 0.84, *P* = 7 × 10^−14^) as well as *STAT2* (ref. ^29^) (ρ = 0.91, *P* = 6 × 10^−20^) but not to *IRF3* or *IFNA2* (both *P* > 0.05) transcripts. In contrast to *IFNA2*, but in agreement with our multivariable logistic regression models for mortality, *IFNB1* levels were most strongly correlated to *IRF7* and *TLR7* plasmacytoid dendritic cell (pDC)-specific type I IFN drivers as well as inflammatory targets, such as *IL6R* (Fig. 4c, lower panel). Similar to *IFNB1*, we found that *IRF3* transcript levels were also able to predict mortality in residents (Fig. 4d; Kaplan–Meier curve, *P* = 0.0030). In addition, classification of nursing home residents according to *IFNB1* levels demonstrated a significant link with lower IRF3 expression as well as higher viral replication and apoptosis, providing a possible molecular and cellular mechanism of action of IFN-β.

**Extended Data Fig. 4:**
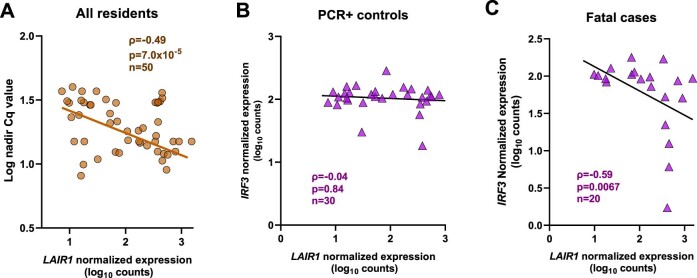
*LAIR1* expression (a surrogate marker for anti-type I IFN neutralizing antibodies) correlates with *IRF3* expression in fatal cases only. (**a**) *LAIR1* expression (nasal mucosa, quantified by nCounter digital transcriptomics) correlates positively with peak SARS-CoV-2 viral load (that is negatively with nadir Cq value) across all residents. (**b**) *LAIR1* expression does not correlate with *IRF3* expression (nasal mucosa) in PCR-positive residents. (**c**) *LAIR1* expression correlates negatively with *IRF3* expression (nasal mucosa) in fatal COVID-19 cases. All correlations Spearman. Source data

In addition to the genetic link to type I IFN signaling, anti-type I IFN neutralizing antibodies have been shown by several groups to be an additional risk factor for life-threatening COVID-19 (refs. ^30–33^). Because no serum samples were available from the fatal cases, we cross-examined our fatal COVID-19 immune gene signature with the LAIR1 biomarker recently described by van der Wijst et al.^33^ as strongly correlated to anti-IFN auto-antibodies. Confirming its antagonistic role in IFN/antiviral signaling, *LAIR1* level was positively correlated with peak viral load (nadir Cq value, *P* = 7.0 × 10^−5^, ρ = −0.49, *n* = 50; Extended Data Fig. 4). Similar to peak viral load (Fig. 2 and Tables 2 and 3), *LAIR1* transcript levels were not able to predict survival in this cohort (data not shown). However, we found that *LAIR1* transcript level was significantly and negatively correlated with *IRF3* (the major upstream driver of type I IFN production in epithelial cells) in fatal cases (ρ = −0.59, *P* = 0.0067, *n* = 20), whereas no significant correlation was observed for matched PCR-positive controls (ρ = −0.037, *P* = 0.84, *n* = 30; Extended Data Fig. 4). This demonstrated a major difference in the type I IFN pathway regulation between fatal cases and controls, probably more pronounced in the epithelial cells of the upper airway mucosa, in agreement with Zhang et al.^34^.

Taken together, cross-examination of published transcriptomic and genomic pre-vaccine fatal COVID-19 signatures highlights the unique innate and adaptive immune signature observed in post-vaccination fatal COVID-19 in nursing home residents.

## Discussion

We comprehensively studied three large outbreaks in Belgian nursing homes with high fatality ratios (20–35%), which resulted in several epidemiologically and clinically relevant insights into the ongoing ‘arms race’ between vaccines and SARS-CoV-2 variants of concern. First, whole-genome phylogenetic analyses indicated that each outbreak stemmed from a single introduction event, although with different variants (Delta, Gamma and Mu). Second, our study confirms previous reports of the independent relationship of older age and male sex with fatal COVID-19, yet is the first to associate Gamma and Mu variants and late onset of PCR positivity with fatal post-vaccination COVID-19 among older adults. Our findings evoke that even non-dominant variants of concern (Gamma) or variants of interest (Mu) can result in high mortality, similar to the dominant variant of concern (Delta at the time of this study, May–August 2021) in specific high-risk settings. Third, environmental sampling revealed that SARS-CoV-2 could be detected in aerosol samples of common spaces (used by either residents or staff) up to 52 d after the initial infection. Fourth, gene expression profiling of nasopharyngeal swabs identified candidate immunological (*IFNB1* and *IRF3*) and virological (*ORF7A* and *ACE2*) biomarkers for early monitoring of post-vaccine breakthrough cases in high-risk older adults, which might not be limited to nursing homes.

Indeed, increased *IFNB1* transcript levels are highlighted as a significant independent predictor of fatal post-vaccination COVID-19, extending our previous findings in critical COVID-19 (ref. ^19^). Although IFN-β therapy was beneficial in small phase 2 clinical trials^35,36^, subsequent larger trials identified no benefit^37^ or even an association with a longer ICU stay^38^, thus underscoring our previous findings on endogenous IFN-β expression in ICU patients^19^. As previously proposed^39,40^, these apparently conflicting effects of type I IFN can be explained by a two-phase model, in which early IFN results in antiviral protection^41^, whereas late IFN exerts a deleterious pro-inflammatory effect. In support of this hypothesis, type I IFN scores were strongly correlated to *STAT2* levels (Fig. 4c, ρ = 0.91, *P* = 6.8 × 10^−20^), for which our group previously demonstrated a simultaneous antiviral and pathogenic in vivo role in a COVID-19 hamster model^29^. In addition, we found that *IFNB1* transcripts were strongly correlated (ρ = 0.84, *P* = 6.8 × 10^−17^) to IL-6 receptor (the target of tocilizumab) expression. Thus, our study suggests IL6/IL6R signaling as a plausible ‘downstream’ therapeutic target in *IFNB1*-overexpressing patients with COVID-19, which should be investigated in future clinical trials.

Regarding the clinical use of transcriptomic biomarkers in COVID-19, only nCounter technology was able to reliably detect *IFNB1* as well as other low-abundance transcripts (*MASP2* and *THY1)* when compared to single-cell RNA sequencing (RNA-seq) analysis of both nasal mucosa^23^ and blood^24^ (data not shown). Moreover, 10 cytokine transcripts found to be overexpressed in fatal cases by nCounter (*IFNA1*, *IFNA2*, *IFNB1*, *IL2*, *IL3*, *IL17B*, *IL17F*, *IL20*, *IL21* and *IL26*) were undetectable or extremely low in several single-cell RNA-seq datasets^22–24^. In addition, only a small subset of these cytokines has been reproducibly detected at the protein level as biomarkers of COVID-19 disease severity and mortality, as evidenced by a recent meta-analysis^42^. In addition, this study also found that nCounter technology outperformed conventional qPCR (Fig. 2a) for virological monitoring of nasopharyngeal swabs to instruct COVID-19 clinical management.

We would like to highlight that, due to the challenging circumstances of the outbreaks (sudden high mortality, extremely high work burden on staff with emergency measures and quarantine, closing of the nursing homes for all visitors and family members not allowed to visit terminally ill residents), all our research analyses (qPCR, SARS-CoV-2 WGS, phylogenetics and immune gene expression profiling) were limited to the diagnostic samples (nasal swabs). Additional (blood) samples for antibody or genetic testing in the fatal cases were logistically and ethically not possible. However, a cross-comparison with published (pre-vaccine) transcriptomic^22^ and genomic^28^ signatures for fatal and life-threatening COVID-19 revealed a surprising dichotomy between IRF3-mediated IFN/antiviral signaling and IR7-mediated IFN/antiviral signaling, which our data suggest as ‘protective’ versus ‘deleterious’, respectively (Fig. 4). Of interest, this dichotomy also provides a possible molecular and cellular mechanism of action for IFN-β, linking IRF7/TLR7 overactivation in pDCs to IL-6R-mediated inflammation, triggering the destruction of IRF3-expressing epithelial cells through apoptosis (Fig. 4), similar to previous findings^43–45^. Notably, a recent study, published during the reviewing process of our study, confirmed the significant link between IFN-β-induced transcriptomic changes and severe COVID-19 in the aging brain^46^. Moreover, the contrasting antiviral and/or pro-apoptotic effects of IFN-β versus IFN-α were shown previously in other pathologies^44–48^. Importantly, anti-IFN-β neutralizing antibodies are infrequent (≤1% of critical COVID-19), in contrast to anti-IFN-α or anti-IFN-ω antibodies, which occur in up to 20% of older patients and fatal COVID-19 (ref. ^49^). Taken together, our findings reveal a need to refine the ‘generic’ type I IFN response (easily quantified by nCounter digital transcriptomics, this study and refs. ^50,51^ or qPCR arrays^41,52^), according to subtypes (IFN-α, IFN-β and IFN-ω), cellular context (epithelial cells and pDCs) and upstream signaling (IRF3 versus IRF7) to accurately predict ‘protective’ versus ‘deleterious’ clinical outcomes.

Finally, our finding of increased viral receptor ACE2, enhanced intracellular viral replication and later onset of PCR positivity in fatal cases hints at a therapeutic window for early antiviral therapy at the start of an outbreak, supported by the recent availability of effective oral antivirals^53–58^. The significantly higher mortality with late onset of infection (PCR positivity >7 d) was observed in each of the three outbreaks (Fig.1 and Extended Data Figs. 1 and 2). We hypothesize that this increased mortality might be due to a higher infectious dose, linked to the exposure to multiple concomitant viral shedders, as compared to early infections. This hypothesis is supported by our demonstration of prolonged detection of SARS-CoV-2 by aerosol PCR in several common rooms of each nursing home. Thus, our study indicates that biomarker-guided clinical trials evaluating the role of early antiviral therapy during post-vaccination nursing home outbreaks, and conceivably also among susceptible community-dwelling older adults, are warranted.

Limitations of this study include missing demographic (8.4% of 657), clinical (2.4% of 620) and vaccination (26.5% of 574) data, although no data were missing for fatal cases. Due to the unpredictable and sudden onset of these large-scale COVID-19 outbreaks in nursing homes, no baseline serum samples were available before the three outbreaks, nor from fatal cases, to compare the levels of vaccine-elicited SARS-CoV-2 neutralizing antibodies or anti-IFN type I auto-antibodies. Moreover, the observational nature of the study and the heterogeneity among three outbreaks (three different variants in nursing homes with different characteristics) might result in residual confounding factors, although the vaccination rates were highly similar among the nursing homes (Supplementary Table 1), and nursing home size did not predict COVID-19 mortality in our study (Supplementary Table 6). Lastly, we did not have specific data on staff pandemic preparedness and population incidence of COVID-19 in the surrounding population, which Suñer et al.^4^ identified as major predictors of (pre-vaccine) COVID-19 mortality in a large retrospective study of Spanish nursing homes. A major strength of this study is the simultaneous vaccination of residents in each nursing home (prioritized in the national vaccination campaign) and the defined onset (outbreaks) of SARS-CoV-2 infections, thus eliminating any possible bias in waning vaccine efficacy between fatal and non-fatal cases. Because this study was performed before vaccination booster doses were offered to older adults in Belgium (starting in September 2021), the risk factors identified herein might not be directly applicable in (recently) boosted older adult populations but remain highly relevant in the global context, in which currently only 63% of people have received an initially full vaccination protocol (two doses), and only 33% have received a booster dose^59^, as exemplified by recent high Omicron COVID-19 mortality among unvaccinated older adults in Hong Kong^60^.

In conclusion, high case fatality ratios in susceptible older adults can be observed with various SARS-CoV-2 variants—that is, Delta, Gamma and Mu. Broad immunovirological profiling of nasal mucosa by nCounter transcriptomics allowed prediction of fatal COVID-19 in diagnostic samples, whereas standard qPCR viral load quantification did not. The best predictive models for mortality comprised *IFNB1* or age, viral *ORF7a* and *ACE2* receptor transcripts, whereas comparison with pre-vaccine fatal COVID-19 signatures uncovered a unique *IRF3* low/*IRF7* high immune signature in post-vaccine fatal COVID-19 outbreaks. A multi-layered strategy including environmental sampling, immunomonitoring and early antiviral therapy should be considered to prevent post-vaccination COVID-19 mortality in nursing homes.

## Methods

### Data collection

Demographic and clinical characteristics, including comorbidities, were compiled from health records provided by the individual nursing homes. The primary outcome was COVID-19-related death, as defined by WHO criteria^16^. All residents, as well as the large majority of staff members, received the BNT162b2 (Comirnaty (Pfizer)) vaccine. This work was framed within the role of the National Reference Centre for Respiratory Pathogens UZ/KU Leuven (as defined by the Royal Decree of 9/2/2011), as approved by the UZ/KU Leuven ethical committee for research (S66037). No written informed consent was obtained for the use of human data and samples; all individuals involved were orally informed of the setup, context and objectives of the study, and all individuals provided oral consent.

### Quantification of viral loads

Consecutive screening events were organized in all three nursing homes, first testing symptomatic individuals, followed by collective and repeated testing after the identification of a positive case. Next to nasopharyngeal swabs of residents and staff, aerosol samples were collected using the AerosolSense instrument (Thermo Fisher Scientific). After RNA extraction, samples were tested by the TaqPath COVID-19 CE-IVD RT–PCR kit (Thermo Fisher Scientific). More details can be found in the Supplementary Methods.

### WGS and phylogenetic analyses

Samples with a sufficiently high viral load (>1,000 copies per milliliter) were subjected to WGS using the ARTIC Network protocol version 3.17 (ref. ^61^) or as described by Freed et al.^62^ and sequenced with Oxford Nanopore Technologies ARTIC library preparation. Complete sequences were recovered using the ARTIC analysis pipeline and typed using Pangolin and NextClade. Specifically, and to investigate if those outbreaks could have been induced by multiple introduction events in the nursing home, we aimed to contextualize the position of those infectious cases in a more global phylogenetic tree built from the analysis of an alignment made of (1) the viral genomes collected in the considered nursing home and sequenced in the context of the present study as well as (2) the genomic sequences of the same variant available for Belgium at the time of the outbreak and (3) a subtree of the European Nextstrain build containing all the genomic sequences of that variant at the time of the outbreak. A time-calibrated maximum likelihood phylogenetic tree was constructed using IQ-TREE version 2.0.3.19 (ref. ^63^) (GTR model)^64^ and TreeTime version 0.8.4.22 (ref. ^65^). Extended protocols are available in the Supplementary Methods.

### Immunovirological profiling by digital transcriptomics (nCounter)

To identify immune and viral risk factors, 600-plex target profiling was performed by digital nCounter transcriptomics (NanoString) in a subset of residents (*n* = 60). RNA was extracted from nasopharyngeal swabs as described above and used for hybridization to pre-specified Human Immunology V2 and customized SARS-CoV-2 panels, as described previously^19–21^. Pathway score analyses and cell type deconvolution were performed using nSolver software (NanoString). Details on the gene lists and pathways are provided in Supplementary Table 5.

### Analysis of publicly available RNA-seq data

Bulk RNA-seq data from both nasopharyngeal and blood samples, as well as corresponding gene signatures of fatal versus non-fatal COVID-19 in hospitalized patients, were obtained from Lee et al.^22^. Single-cell RNA-seq data^23^ of nasopharyngeal samples of 19 patients with COVID-19 (eight moderate and 11 critical, according to WHO classification) and five healthy controls were obtained from 10.6084/m9.figshare.12436517; single-cell RNA-seq data from peripheral blood mononuclear cell (PBMCs) of patients with COVID-19 were obtained from http://www.covidcellatlas.com/ (ref. ^24^).

#### Statistics and reproducibility

Owing to the nature of the study (nationwide comprehensive mapping of high-fatality SARS-CoV-2 outbreaks in nursing homes), no statistical methods were used to pre-determine sample sizes, and data collection and analysis were not performed blinded to the clinical outcome (fatal COVID-19). Demographic and clinical data (COVID symptoms, detailed pre-existing comorbidities, clinical outcome from all residents and pre-COVID pharmacological data; the level of detail differed per nursing home) were collected from electronic health records provided by the nursing homes and hospitals. Missing data were not imputed, and only individuals with all available parameters respective to the specific model were included. Stepwise logistic regression was used to identify risk factors for fatal COVID-19, and the best model was selected using cAIC. Kaplan–Meier estimates of survival were calculated up to 60 d after the first SARS-CoV-2 PCR-positive case in each nursing home outbreak. Selected predictors were confirmed by Cox proportional hazard regression, defining survival in days since PCR diagnosis. In sensitivity analyses, only fully vaccinated (defined as two BNT162b2 doses received at least 14 d before the start of the outbreak) and PCR-positive residents were included. Because transcriptomic data did not follow a normal distribution (determined by Kolmogorov–Smirnov test), non-parametric Kruskal–Wallis test (with FDR correction for multiple testing) was used to compare three groups (PCR-negative, PCR-positive and fatal cases) and Mann–Whitney test for two groups (fatal versus non-fatal). Pathway scores were normally distributed and analyzed using a *t*-test (with Welch’s correction when needed, fatal versus non-fatal groups). All statistical tests were two-sided.

### Reporting summary

Further information on research design is available in the Nature Portfolio Reporting Summary linked to this article.

## Supplementary information

Supplementary Information Supplementary Information and Supplementary Methods

Reporting Summary

Supplementary Tables Supplementary Tables 1–6

**Supplementary Data**

## Data Availability

All data are included in the manuscript, Source Data files and supplementary files. Demographic, clinical and nCounter data are available from the authors upon reasonable request, due to privacy protection. All Source Data and code related to phylogenetic analysis (Fig. 1 and Extended Data Figs. 2 and 3) are available for download at https://www.zidu.be/SI_data.zip. The following GISAID IDs corresponding to SARS-CoV-2 genomes were generated as part of this study: EPI_ISL_2289002, EPI_ISL_2301430, EPI_ISL_2304141, EPI_ISL_2304143, EPI_ISL_2348574-78, EPI_ISL_2348580-86, EPI_ISL_2348587-92, EPI_ISL_2626083-96, EPI_ISL_2864473-74, EPI_ISL_2864478, EPI_ISL_2864483, EPI_ISL_2864485, EPI_ISL_2864489, EPI_ISL_2864573-76, EPI_ISL_2864707-10, EPI_ISL_2864714-15, EPI_ISL_2864717-21, EPI_ISL_2886237, EPI_ISL_3118412-26, EPI_ISL_4007338, EPI_ISL_4008034, EPI_ISL_4008052, EPI_ISL_4348705, EPI_ISL_4348711, EPI_ISL_4348959, EPI_ISL_4354278, EPI_ISL_4358318, EPI_ISL_4571448-51 and EPI_ISL_5349110.

## Source data

Source Data Statistical source data for Figs. 2–4
Source Data Source data for Extended Data Figs. 3 and 4

## Code availability

All code necessary to reproduce the findings of this study is provided in the Supplementary Information: https://www.zidu.be/SI_data.zip.
