# Supplementary material for: Immunovirological and environmental screening reveals actionable risk factors for fatal COVID-19 during post-vaccination nursing home outbreaks
Source: Nat Aging. 2023 May 22;3(6):722–33. doi: 10.1038/s43587-023-00421-1 (PMC10275758; doi:10.1038/s43587-023-00421-1)
Supplement: Supplementary file 1 — Supplementary Information and Supplementary Methods [file 43587_2023_421_MOESM1_ESM.pdf]

nature aging

Article https://doi.org/10.1038/s43587-023-00421-1

Immunovirological and environmental screening reveals actionable risk factors for fatal COVID-19 during post-vaccination nursing home outbreaks

In the format provided by the authors and unedited

nature aging

Article https://doi.org/10.1038/s43587-023-00421-1

Immunovirological and environmental screening reveals actionable risk factors for fatal COVID-19 during post-vaccination nursing home outbreaks

In the format provided by the authors and unedited

Supplementary Information

All Source data and code related to phylogenetic analysis (Fig. 1, Suppl. Fig.2-3) is available for download at https://www.zidu.be/SI_data.zip.

Supplementary Methods

Quantification of viral loads

Aerosol samples were collected using the AerosolSense instrument (Thermo Fisher Scientifics), sampling 200 liters of room air per minute, for a total of 18-26h. Viruses are trapped on the collection substrate of a removable sample cartridge (Thermo Fisher Scientifics), followed by lysis in transport buffer (DNA/RNA Shield, Zymo Research). RNA extraction was performed using the DEXR-15-LM96 kit for automated extraction (Diagenode, Seraing, Belgium) with 350 µl sample input. Samples were spiked with a purified MS2 bacteriophage as internal control according to the manufacturer's instructions (Thermo Fisher Scientific, A47817). Extracted RNA was eluted from magnetic beads in 50 µl of UltraPure DNase/RNase free distilled water. RT-PCR testing was performed with the TaqPath COVID-19 CE-IVD RT-PCR kit (Thermo Fisher Scientific). Results were analyzed using the FastFinder analysis software (Ugentec, Belgium) and expressed as a quantification cycle (Cq) for the ORF1ab, N, and S gene targets. Samples were reported negative when all viral gene targets were above a Cq value of 37 and when the MS2 internal control Cq value was lower than 27.5.

Whole-genome sequencing and phylogenetic analyses

Samples with a sufficiently high viral load (>1000 RNA copies/ml) were analyzed using whole-genome sequencing. An automatic RNA extraction was performed using the MagMAx Viral / Pathogen kit II (MVPII) (Thermo Fisher Scientific, A48383) with 200 µl sample input. The genomes were amplified following the ARTIC network protocol V3 or V4 [1] or as described by Freed *et al.*[2]. After clean-up of the amplicons, libraries were prepared using the SQK-LSK109+EXP-NBD196 ligation sequencing kit from Oxford Nanopore Technologies. Subsequently, the libraries were quantified, and sequencing was performed on a GridION platform using MinKNOW's built-in basecalling, demultiplexing and adapter trimming. Sequencing runs were processed using the ARTIC analysis pipeline and custom scripts. Full-length genome sequences accompanied with metadata were submitted to GISAID (see list of accession IDs). SARS-CoV-2 lineage assignments were derived using the Pangolin tool and Nextclade (https://clades.nextstrain.org/). The following GISAID IDs corresponding to SARS-CoV-2 genomes were generated as part of this study: EPI_ISL_2289002, EPI_ISL_2301430, EPI_ISL_2304141, EPI_ISL_2304143, EPI_ISL_2348574-78, EPI_ISL_2348580-86, EPI_ISL_2348587-92, EPI_ISL_2626083-96, EPI_ISL_2864473-74, EPI_ISL_2864478, EPI_ISL_2864483, EPI_ISL_2864485, EPI_ISL_2864489, EPI_ISL_2864573-76, EPI_ISL_2864707-10, EPI_ISL_2864714-

15, EPI_ISL_2864717-21, EPI_ISL_2886237, EPI_ISL_3118412-26, EPI_ISL_4007338, EPI_ISL_4008034, EPI_ISL_4008052, EPI_ISL_4348705, EPI_ISL_4348711, EPI_ISL_4348959, EPI_ISL_4354278, EPI_ISL_4358318, EPI_ISL_4571448-51, EPI_ISL_5349110.

To analyze the evolutionary relationships among infectious cases, we performed a distinct phylogenetic analysis, each time focused on one of three variants involved in a specific nursing home outbreak. For each nursing home, the phylogenetic analysis was based on an alignment made of (i) the viral genomes collected in the considered nursing home and sequenced in the context of the present study, as well as (ii) the genomic sequences of the same variant available for Belgium at the time of the outbreak, and (iii) a subtree of the European Nextstrain build containing all the genomic sequences of that variant at the time of the outbreak. Other sequences ancestrally related in the Nextstrain build to the variants under study were included to ensure sufficient temporal signal for a molecular clock calibration. To construct the multiple sequence alignments, we mapped each data set against the reference sequence EPI_ISL_406801 using minimap2 v2.20-r1061 [6] and trimmed the data to positions 265-29,674 to mask out 3' and 5' untranslated regions. To obtain a maximum-likelihood phylogeny, we ran IQ-TREE 2.0.3 [3] using a general time-reversible (GTR) model [4] of nucleotide substitution with empirical base frequencies and four free site rate categories,[7] which was selected as the optimal model using IQ-TREE's ModelFinder tool. We then time-calibrated the phylogeny using TreeTime 0.8.4 [5] and set to filter samples that deviated more than four interquartile ranges from a root-to-tip regression.[8] The final alignments were made of 1728 genomic sequences for nursing home A (Delta; 1128 from Belgium, 58 from the nursing home), 2341 for nursing home B (Gamma; 2036 from Belgium, 6 from the nursing home), and 289 for nursing home C (Mu; 28 from Belgium, 24 from the nursing home).

Supplementary References:

1. Quick J. nCoV-2019 sequencing protocol. Available on https://artic.network/ncov-2019.

2. Freed NE, Vlkova M, Faisal MB, Silander OK. Rapid and inexpensive whole-genome sequencing of SARS-CoV-2 using 1200 bp tiled amplicons and Oxford Nanopore Rapid Barcoding. Biology Methods and Protocols 2020;5.

3. Minh BQ, Schmidt HA, Chernomor O, Schrempf D, Woodhams MD, von Haeseler A, Lanfear R. IQ-TREE 2: new models and efficient methods for phylogenetic inference in the genomic era. Mol. Biol. Evol 2020;37(5):1530–1534.

4. Tavaré S. Some probabilistic and statistical problems in the analysis of DNA sequences. Lectures Math. Life Sci 1986;17:57–86.

5. Sagulenko P, Puller V, Neher RA. TreeTime: Maximum-likelihood phylodynamic analysis. Virus Evol 2018;4:vex042.

6. Heng L. Minimap2: pairwise alignment for nucleotide sequences. Bioinformatics 2018;34(18):3094-3100.

7. Yang Z. A space-time process model for the evolution of DNA sequences. Genetics 1995;139:993–1005.

8. Dellicour S, Durkin K, Hong SL, et al. A phylodynamic workflow to rapidly gain insights into the dispersal history and dynamics of SARS-CoV-2 lineages. Mol. Biol. Evol 2021;38(4):1608-1613.
