# Supplementary material for: Immunovirological and environmental screening reveals actionable risk factors for fatal COVID-19 during post-vaccination nursing home outbreaks
Source: Nat Aging. 2023 May 22;3(6):722–33. doi: 10.1038/s43587-023-00421-1 (PMC10275758; doi:10.1038/s43587-023-00421-1)
Supplement: Supplementary file 2 — Reporting Summary [file 43587_2023_421_MOESM2_ESM.pdf]

nature portfolio

Corresponding author(s): Johan Van Weyenbergh

Last updated by author(s): 2023/01/08

Reporting Summary

Nature Portfolio wishes to improve the reproducibility of the work that we publish. This form provides structure for consistency and transparency in reporting. For further information on Nature Portfolio policies, see our Editorial Policies and the Editorial Policy Checklist.

Statistics

For all statistical analyses, confirm that the following items are present in the figure legend, table legend, main text, or Methods section.

n/a	Confirmed	
☐	☒	The exact sample size (*n*) for each experimental group/condition, given as a discrete number and unit of measurement
☐	☒	A statement on whether measurements were taken from distinct samples or whether the same sample was measured repeatedly
☐	☒	The statistical test(s) used AND whether they are one- or two-sided<br>*Only common tests should be described solely by name; describe more complex techniques in the Methods section.*
☐	☒	A description of all covariates tested
☐	☒	A description of any assumptions or corrections, such as tests of normality and adjustment for multiple comparisons
☐	☒	A full description of the statistical parameters including central tendency (e.g. means) or other basic estimates (e.g. regression coefficient) AND variation (e.g. standard deviation) or associated estimates of uncertainty (e.g. confidence intervals)
☐	☒	For null hypothesis testing, the test statistic (e.g. *F*, *t*, *r*) with confidence intervals, effect sizes, degrees of freedom and *P* value noted<br>*Give P values as exact values whenever suitable.*
☒	☐	For Bayesian analysis, information on the choice of priors and Markov chain Monte Carlo settings
☒	☐	For hierarchical and complex designs, identification of the appropriate level for tests and full reporting of outcomes
☒	☐	Estimates of effect sizes (e.g. Cohen's *d*, Pearson's *r*), indicating how they were calculated

Our web collection on statistics for biologists contains articles on many of the points above.

Software and code

Policy information about availability of computer code

Data collection	Nextstrain was used to sample European genomic sequences and other sequences ancestrally related to the variants under study.
Data analysis	All software used is open-source and detailed in the Supplementary Methods. Software/tools used: Nextstrain (SARS-CoV-2 Workflow, development version), Pangolin and Nextclade (https:// clades.nextstrain.org/), ARTIC version 3 and 4, IQ-TREE 2.0.3, TreeTime 0.8.4 As described in the Code Availability statement, all code necessary to reproduce the findings of this study is provided in the Supplementary Information.

For manuscripts utilizing custom algorithms or software that are central to the research but not yet described in published literature, software must be made available to editors and reviewers. We strongly encourage code deposition in a community repository (e.g. GitHub). See the Nature Portfolio guidelines for submitting code & software for further information.

Data

Policy information about availability of data

All manuscripts must include a data availability statement. This statement should provide the following information, where applicable:

- Accession codes, unique identifiers, or web links for publicly available datasets
- A description of any restrictions on data availability
- For clinical datasets or third party data, please ensure that the statement adheres to our policy

All data are available from the authors upon request, GISAID IDs corresponding to full SARS-CoV-2 genomes generated as part of this study are listed in Supplementary Methods.

Human research participants

Policy information about studies involving human research participants and Sex and Gender in Research.

Reporting on sex and gender	Only biological sex was reported, since this was known as an important parameter of COVID-19 mortality.
Population characteristics	The population consisted of nursing home residents, characterized by older age, predominant female sex and high burden of comorbidities (summary statistics per nursing home are given in Suppl. Table 4.).
Recruitment	All Belgian nursing homes were monitored for post-vaccine COVID19 outbreaks and all residents from nursing homes with high case fatality (>10%) were recruited
Ethics oversight	This work was framed within the role of the National Reference Centre for respiratory pathogens UZ/KU Leuven (as defined by the Royal Decree of 09/02/2011), as approved by the UZ/KU Leuven Ethical committee for research (S66037).

Note that full information on the approval of the study protocol must also be provided in the manuscript.

Field-specific reporting

Please select the one below that is the best fit for your research. If you are not sure, read the appropriate sections before making your selection.

☒ Life sciences ☐ Behavioural & social sciences ☐ Ecological, evolutionary & environmental sciences

For a reference copy of the document with all sections, see nature.com/documents/nr-reporting-summary-flat.pdf

Life sciences study design

All studies must disclose on these points even when the disclosure is negative.

Sample size	No sample size calculation was performed, all outbreaks were monitored at national level.
Data exclusions	No data were excluded in the study.
Replication	This was an observational study, all Belgian nursing homes with high post-vaccine COVID-19 case-fatality (>10%) were included (3 in total).
Randomization	Allocation was not random, all available samples were analyzed in each nursing home. Known (age, sex, comorbidities, viral load) and novel covariates were characterized by univariate and multivariate logistic regression.
Blinding	Investigators were not blinded to group allocation during data collection and analysis, since all data needed to be reported as part of the SARS-CoV-2 surveillance as National Reference Centre.

Reporting for specific materials, systems and methods

We require information from authors about some types of materials, experimental systems and methods used in many studies. Here, indicate whether each material, system or method listed is relevant to your study. If you are not sure if a list item applies to your research, read the appropriate section before selecting a response.

Materials & experimental systems

n/a	Involved in the study
☒	☐ Antibodies
☒	☐ Eukaryotic cell lines
☒	☐ Palaeontology and archaeology
☒	☐ Animals and other organisms
☒	☐ Clinical data
☒	☐ Dual use research of concern

Methods

n/a	Involved in the study
☒	☐ ChIP-seq
☒	☐ Flow cytometry
☒	☐ MRI-based neuroimaging
